# A Prospective Observational Study Analyzing the Analgesic Efficacy of Caudal Block and Nerve Stimulator-Guided Pudendal Nerve Block in Children Undergoing Hypospadias Repair

**DOI:** 10.7759/cureus.44649

**Published:** 2023-09-04

**Authors:** Nitin Hayaran, Parul Kaushik, Sangeeta Yadav, Anga Hage

**Affiliations:** 1 Anesthesiology, Lady Hardinge Medical College (LHMC), New Delhi, IND; 2 Anesthesiology and Critical Care, Atal Bihari Vajpayee Institute of Medical Sciences (ABVIMS) and Dr. Ram Manohar Lohia (RML) Hospital, New Delhi, IND; 3 Anesthesiology and Critical Care, Lady Hardinge Medical College (LHMC), New Delhi, IND

**Keywords:** caudal anesthesia, pediatric, postoperative pain, nerve block, hypospadias

## Abstract

Introduction: Hypospadias repair is a common pediatric surgery. While caudal block (CB) is the most widely used procedure for analgesia, pudendal nerve block (PNB) can be utilized as an alternative. We evaluated the postoperative analgesic efficacy of CB and PNB in children undergoing hypospadias repair.

Methods: In this prospective observational study, we evaluated 101 patients who received standard general anesthesia along with CB or PNB. Postoperative pain score (Face, Legs, Activity, Cry, and Consolability {FLACC} score) along with the total number of analgesic doses and the total amount of analgesic drugs consumed within 24 hours were noted. Time to first rescue analgesia, post-block penile length and midshaft circumference, surgeon satisfaction score, and postoperative complications were also evaluated.

Results: Out of 101 patients observed, 50 received CB, and 51 received PNB. At 24-hour interval, the median FLACC score in CB was 6, while in the PNB, it was 4 (p<0.001). None of the patients who were given PNB received more than three doses (p<0.001), and the average consumption of analgesic drugs within 24 hours was significantly higher in CB (38.4±4.28) compared to PNB (21.7±6.33) (p<0.0001). The median time to first rescue analgesia in CB was four hours, while in patients receiving PNB, it was eight hours (p<0.001). The increase in penile volume was significantly higher with CB as compared to PNB (p<0.001). Surgeon satisfaction score was found to be better with PNB (p<0.002).

Conclusion: Patients who received PNB had significantly reduced pain scores and analgesic consumption in the first 24 hours post surgery.

## Introduction

Hypospadias is a common congenital malformation of the penis with worldwide prevalence varying from 5.2 to 34.2 per 10000 male births [1-3]. It is attributed to arrest in the embryological development of the urethra, foreskin, and ventral aspect of the penis [4]. Hypospadias is characterized by a proximal urethral opening, which can be located anywhere from the ventral aspect of the glans to the perineum; based on this ectopic urethral meatus, it is classified into distal penile, midshaft, or proximal hypospadias [4]. Surgery is inevitable to correct the defect; hence, it is generally recommended in early childhood for improved function and cosmesis [3]. Surgery is known to be associated with severe pain in the postoperative period, which is not adequately managed by systemic analgesia [5,6]. Caudal block (CB) and dorsal pudendal nerve block (DPNB) are the most commonly performed and widely studied regional analgesia techniques for this surgery [7-10]. DPNB is associated with higher pain scores and increased analgesic consumption when compared to CB [7,10].

CB is one of the most widely used regional techniques for providing both intraoperative and postoperative analgesia. It is simple and easy to perform, reduces surgical stress response, and reduces requirement for systemic analgesia [8,11]. It may lead to complications such as motor block, urinary retention, intravascular injection, and urethral fistula formation in postoperative period [8,12,13]. Even with the use of varying concentrations of different local anesthetics and the addition of various adjuvants, the duration of analgesia with CB remains short, and some adjuvants were associated with complications in the postoperative period [13-19].

Pudendal nerve block (PNB) has been widely performed in the adult population for various pelvic and perianal surgeries [20]. It is mostly performed blind or with the aid of fluoroscopy and nerve stimulation and recently by ultrasound (US) guidance [21-27]. PNB as an alternative analgesic technique following hypospadias repair is found to be effective in reducing perioperative analgesic consumption and providing a prolonged pain-free postoperative period with fewer complications [1,8,22,27]. Hence, the present study was designed to compare the efficacy of postoperative analgesia with CB or PNB following hypospadias repair.

## Materials and methods

This prospective observational study was conducted in our institute on approval from the Ethics Committee for Human Research (LHMC/ECHR/2017/167). After obtaining written informed parental consent, 101 pediatric patients aged 2-10 years, posted for elective hypospadias repair, were enrolled in the study. Patients with spinal anomalies, infection at the local site, known allergy to local anesthetics, preexisting cardiopulmonary diseases, deranged coagulation profile, and revision surgeries and those encountering failure of the block were excluded from the study.

As per standard protocols, all patients received general anesthesia with fentanyl injection (1-2 μg/kg) as premedication; induction was carried out using propofol injection (1.5-2.5 mg/kg) followed by the placement of appropriate-size supraglottic airway device. Anesthesia was maintained with oxygen, nitrous oxide, and sevoflurane (1%-2%) with spontaneous ventilation. After the induction of general anesthesia, as per the experienced anesthesiologist, the patient received either landmark-based CB or nerve stimulator-guided PNB with 0.5 mL/kg of 0.25% bupivacaine with 1 μg/kg fentanyl as an additive.

CB was performed under all aseptic precautions with the patient in lateral decubitus position and legs flexed at the hips. With the help of the landmark method and the loss of resistance technique, the block was administered with a 23 gauge caudal needle.

**Figure 1:**
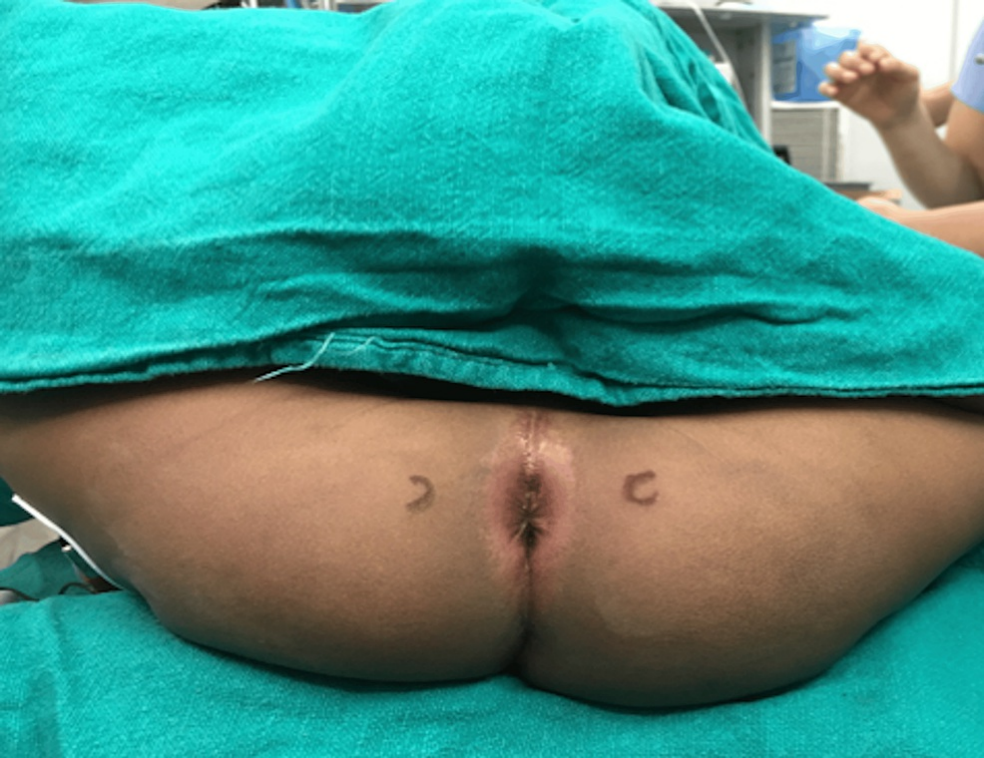
Landmark for pudendal nerve block on either side

PNB was administered in the lithotomy position using nerve stimulator guidance. Two separate injection points were marked at 3 and 9 o’clock positions located midway between the ischial tuberosity and anus on each side (Figure 1).

**Figure 2:**
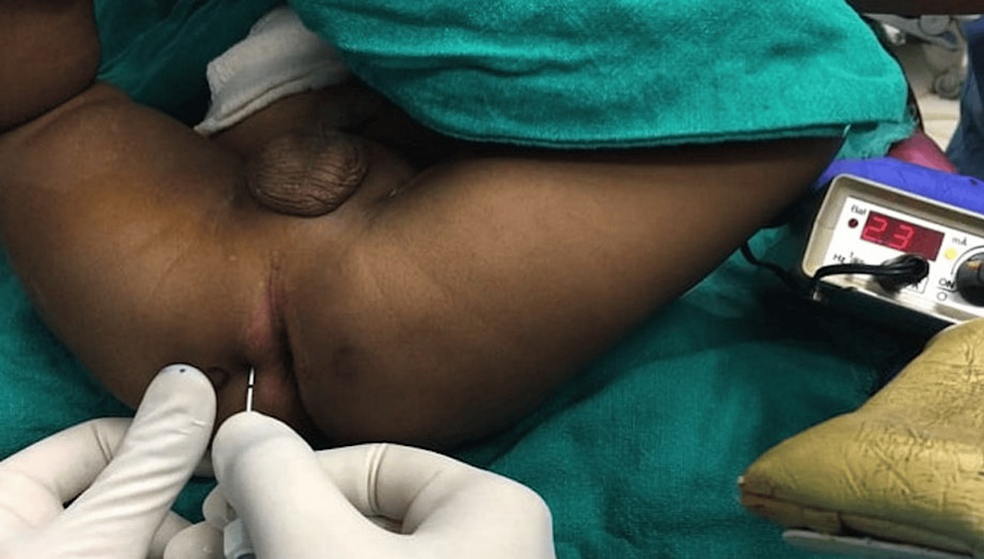
The technique of nerve localization for pudendal nerve block

After the aseptic preparation of the skin, a 5 cm nerve stimulator needle (22-24 gauge Stimuplex® A, 50-100 mm, B. Braun, Melsungen, Germany) was advanced 1.5-3.5 cm perpendicular to the skin using a stimulation current of 2.5-5 mA and 2 Hz (Figure 2). Needle was progressively moved deeper until an up-and-down penile movement was observed; at this moment, stimulation current was decreased to 0.5 mA maintaining contraction, hence conforming the proximity to the nerve. Half the drug was injected at 3 o’clock position and the other half at 9 o’clock position. Patient demographics and total surgical duration were recorded. Penile volume was calculated by measuring the penile length (from the symphysis pubis to the tip) and circumference (midshaft of the penis) before and 10 minutes after both blocks. The Face, Legs, Activity, Cry, and Consolability (FLACC) scale (Table 1) at zero, two, four, eight, 12, and 24 hours in the postoperative period and any postoperative complications such as nausea, vomiting, pruritus, infection, or hematoma at the site of injection were noted in the patient records (PR). A FLACC score of more than 5 was taken as failure of the block and was excluded from the study. A score of more than or equal to 4 was managed with additional analgesia in the form of syrup ibuprofen 10 mg/kg, and if it still remained high (>4), IV tramadol 1 mg/kg was used as rescue analgesic. The total number of doses and the total amount of analgesic consumed in the first 24 hours were noted. Surgeon satisfaction score with respect to operating conditions and postoperative pain was recorded at the end of 24 hours from the operating team, which was the same for all the cases (1, unsatisfied; 2, satisfied; and 3, definitely satisfied).

**Table 1:** FLACC score FLACC: Face, Legs, Activity, Cry, and Consolability

Behavior	0	1	2
Face	No particular expression or smile	Occasional grimace or frown; withdrawn and disinterested	Frequent to constant frown, clenched jaw, and quivering chin
Legs	Normal position or relaxed	Uneasy, restless, and tense	Kicking or legs drawn up
Activity	Lying quietly, moves easily, and normal position	Squirming, shifting back and forth, and tense	Arched, rigid, or jerking
Cry	No cry (awake or asleep)	Moans or whimpers and occasional complaint	Crying steadily, screams or sobs, and frequent complaint
Consolability	Content and relaxed	Reassured by occasional touching, hugging, or being talked to; distractable	Difficult to console or comfort

All data was collected and analyzed statistically using the computer software Statistical Package for Social Sciences (SPSS) version 16 (SPSS Inc., Chicago, IL). The qualitative variables were expressed as frequencies/percentage and compared using chi-square test or Mann-Whitney test. Student’s t-test was used to assess the quantitative variables. A p value of <0.05 was considered statistically significant.

## Results

**Figure 3:**
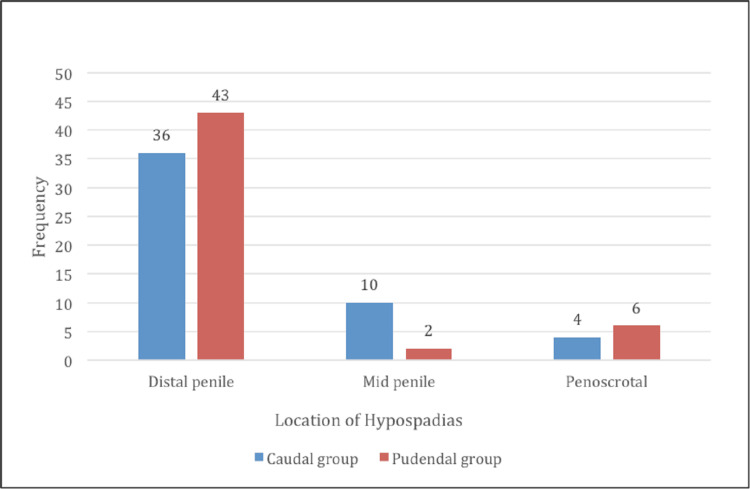
Bar graph depicting the distribution of patients based on the location of hypospadias

The patient’s baseline demographics and clinical characteristics were found to be comparable in both groups. The mean duration (in hours) of surgery in patients receiving CB was 2±0.03, while it was 2.02±0.1 in PNB. Of the 50 patients who received CB, 36 (72%) were operated for distal penile, 10 (20%) for mid-penile, and four (8%) for penoscrotal hypospadias. Of the 51 patients with PNB, 43 (84.3%) were operated for distal penile, two (3.92%) for mid-penile, and six (11.76%) for penoscrotal hypospadias (Figure 3).

**Figure 4:**
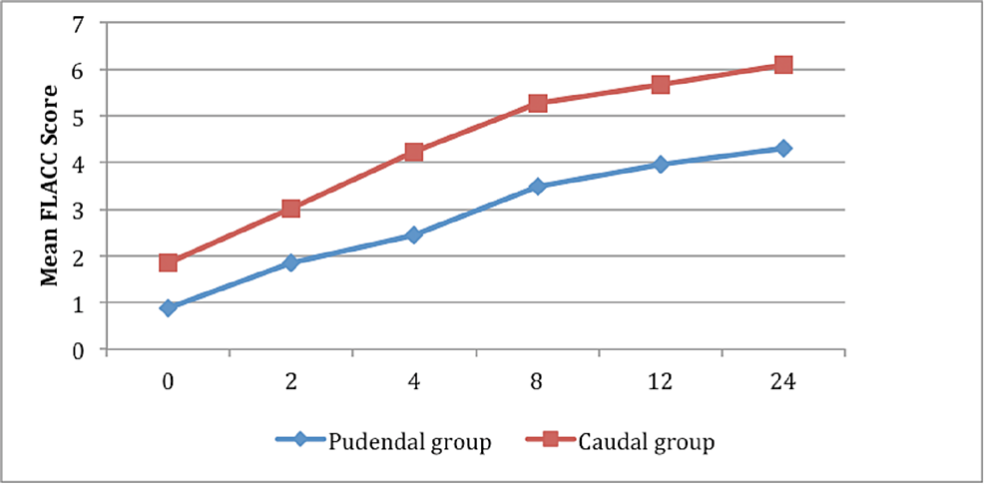
Line diagram demonstrating the mean FLACC score at various time intervals FLACC: Face, Legs, Activity, Cry, and Consolability

Immediately after surgery (zero hours), the median FLACC score in CB was 2, whereas in PNB, it was 0 (p<0.001) (Table 2). Similarly, at two-, four-, eight-, and 12-hour intervals, the median FLACC score was significantly low in those who received PNB (p<0.001). At 24-hour interval, the score in CB was 6, whereas in PNB, it was 4 (P<0.001) (Figure 4).

**Table 2:** FLACC score at various postoperative time intervals IQR, interquartile range; FLACC, Face, Legs, Activity, Cry, and Consolability

FLACC score at hours	Caudal block (n=50)		Pudendal nerve block (n=51)		p value (Mann-Whitney)
	Median	IQR	Median	IQR	
0	2	1-2	0	0-2	<0.001
2	3	2-3	2	2-2	<0.001
4	4	4-5	2	2-3	<0.001
8	5	5-6	3	3-4	<0.001
12	6	5-6	4	4-4	<0.001
24	6	6-6	4	4-5	<0.001

The total number of additional analgesic dose requirement was significantly higher in CB (p<0.001) (Table 3). The median time to first rescue analgesia was four hours with CB, while with PNB, it was eight hours (p<0.001). The 24-hour average analgesic consumption (mg/kg) was significantly higher in CB (38.4±4.28) as compared to PNB (21.7±6.33) (p<0.000) (Table 4).

**Table 3:** Total number of analgesic doses given

Total number of analgesics	Study		Total, n (%)	p value
	Caudal, n (%)	Pudendal, n (%)		<0.001
1	0 (0)	7 (13.72)	7 (6.93)	
2	0 (0)	32 (62.74)	32 (31.68)	
3	12 (24)	12 (23.52)	24 (23.76)	
4	38 (76)	0 (0)	38 (37.62)	
Total	50 (100)	51 (100)	101 (100.0)	

**Table 4:** Mean consumption of analgesic

Average amount of analgesics consumed (mg/kg) in 24 hours	Study				p value
	Caudal block		Pudendal nerve block		
	Mean	SD	Mean	SD	
	38.4	4.28	21.17	6.33	<0.0001

Out of the 50 patients who received CB, 27 (54%) had increase in penile volume 10 minutes after the block, whereas no change in penile volume was observed with PNB (p<0.001).

**Figure 5:**
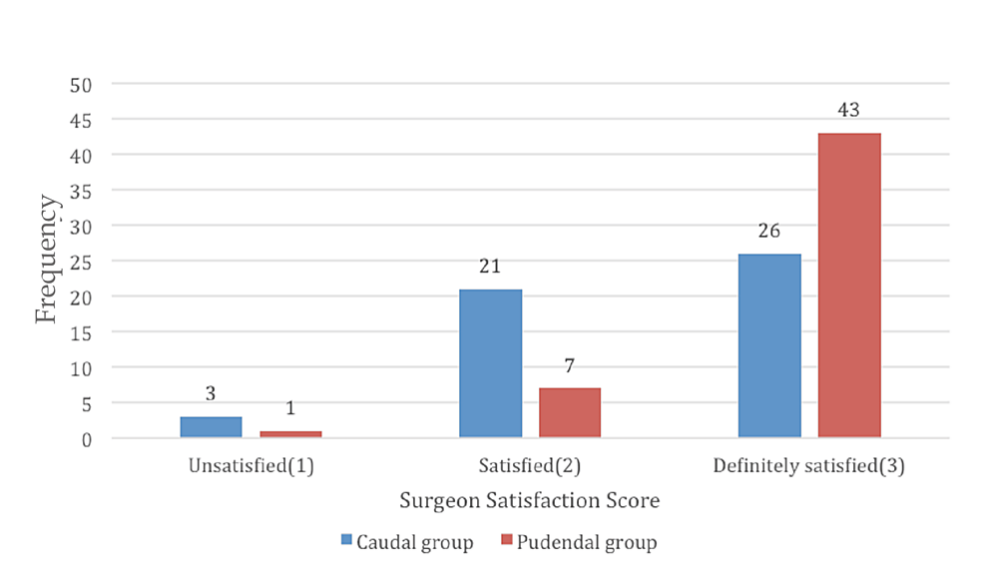
Bar graph depicting surgeon satisfaction score

Surgeons reported higher satisfaction scores in patients with PNB, with a score of 2 in 84.3% of patients as compared to 52% in CB; this observation was found to be highly significant (p<0.002) (Figure 5).

The rate of complication was comparable with both blocks (Table 5).

**Table 5:** Distribution based on postoperative complications

Postoperative complication	Study		Total, n (%)	p value
	Caudal block, n (%)	Pudendal nerve block, n (%)		
Nil	45 (90.0)	49 (96.1)	94 (93.1)	0.307
Nausea	3 (6.0)	2 (3.9)	5 (5.0)	
Vomiting	2 (4.0)	0 (0.0)	2 (2.0)	
Total	50 (100.0)	51 (50.5)	101 (100.0)	

## Discussion

Hypospadias repair is a commonly performed surgery in pediatric patients. Surgery involves creating a tube to increase the length of the urethra so it opens at the tip of the penis. As the surgery involves significant tissue dissection, it is associated with severe pain in the postoperative period especially when managed by systemic analgesics alone [5,6]. Thus, for postoperative analgesia, it is routine to administer regional anesthesia in the form of either caudal block, pudendal nerve block, or dorsal penile nerve block [7,8]. DPNB application is easier but has been associated with complications such as hematoma, edema, and inconsistent analgesia, as it does not cover the ventral surface of the penis, which is supplied by the perineal branch of the pudendal nerve [7,9,10]. All three branches of the pudendal nerve, namely, the dorsal penile nerve, perineal branch, and inferior anal nerve, are blocked at the level of Alcock’s canal by a single nerve stimulator-guided PNB [24].

The results of our study show that PNB had lower pain scores when compared to CB at all assessment intervals in the first 24 hours of the postoperative period. The difference in FLACC score in the two blocks was statistically significant across all postoperative time points, that is, immediately after surgery (zero hours) and two-hour, four-hour, eight-hour, 12-hour, and 24-hour intervals.

There are few studies on the direct comparison of CB to PNB in patients undergoing hypospadias surgery [1,8,22,26,27]. The study conducted by Naja et al. [1] was very similar to our study, as the pain scores in the pudendal group were statistically lower compared to the caudal group at 12 hours, 18 hours, and 24 hours (p<0.001). In their study, the first evaluation of pain was done at the sixth hour after surgery, which showed a higher pain score in the caudal group than the pudendal group, but it was not statistically significant. They used clonidine 1 μg/kg in both groups, which might have made the blocks more dense for a longer period, whereas we gave fentanyl as per protocol in both blocks, which has not shown to increase the duration and quality of block in previous studies [28].

In a more recent study, Kendigelen et al. [8] observed similar results in relation to postoperative pain scores in the first 24 hours. Patients receiving PNB had lower pain scores in comparison to those who received CB at all the points of assessment after 30 minutes of surgery, but it became significant after six hours of surgery (p<0.001).

However, a recent study by Choudhry et al. [26] demonstrated that in children less than two years old, both blocks provide comparable intraoperative and 24-hour postoperative pain relief after hypospadias surgery.

In our study, 0.5 mL/kg drug was administered, whereas in the studies mentioned above, the volume of caudal block was 1 mL/kg. Thus, a faster regression of caudal block was expected in our patients. Therefore, the difference in pain scores in our study became statistically significant from the initial evaluation period.

A study by Ahmed et al. [27] demonstrated that US-guided PNB provided significantly prolonged postoperative analgesia, reduced postoperative analgesic requirements, and better parents’ satisfaction as compared to caudal block in pediatric patients undergoing hypospadias surgery with comparative safety profile. They found higher pain score in the caudal group than in the pudendal group at six hours and 12 hours (p value of 0.017 and 0.003, respectively).

In their study, Hecht et al. [22] did not find any difference in pain scores between the two groups in the recovery unit, while further follow-up in ward was not done. The volume and type of local anesthetic along with the additives were not constant in both groups.

Most of the patients with CB received four doses of additional analgesic, whereas with PNB, patients mostly received only two doses of ibuprofen syrup in the first 24 hours. None of the patients with PNB required more than three doses of additional analgesic (p<0.001). None of the patients required tramadol injection with either of the blocks. The mean analgesic consumption of ibuprofen with CB was approximately double the consumption with PNB (p<0.001), which was similar to the study by Naja et al. [1]. Kendigelen et al. [8] gave IV tramadol 1 mg/kg in the first evaluation and paracetamol (15 mg/kg, IV) whenever the pain score was 7 or greater in the postanesthesia care unit (PACU), while ibuprofen (10 mg/kg by mouth) was administered in the ward. In their study, 10 (25%) patients required additional analgesia in the caudal group, while none of the patients in the pudendal group needed analgesia in the PACU. Nearly all of the patients in the caudal group needed additional analgesia at each point of assessment from the sixth hour onward after the surgery. Though the results of the caudal group are similar to our study, the prolonged period of analgesia in the pudendal group in their study may be attributed to more lateral approach for pudendal nerve localization as compared to our approach. The medial and lateral approach for the localization of the pudendal nerve block needs to be further evaluated for the duration of postoperative analgesia.

In their study, Kundra et al. [9] had also observed the duration of analgesia in caudal block with 0.5 mL/kg to be around four hours. In our study, 0.5 mL/kg of 0.25% bupivacaine was effective but had a slightly less duration of postoperative analgesia.

The time to first rescue analgesia with PNB in our study corroborates with those of Naja et al. [1], but the study by Kendigelen et al. [8] showed time to rescue analgesia at 18 hours.

Twenty-seven patients (54%) with CB had an increase in penile volume 10 minutes after the block, whereas none of the patients with PNB showed change in penile volume (p<0.001). Our finding is consistent with the study done by Kundra et al. [9], where the caudal block group showed significant increase in penile volume as compared to the penile block as it causes sympathetic blockade leading to vasodilatation causing the pooling of blood in the penis resulting in penile engorgement and increased intraoperative blood loss [9].

Surgeons were more satisfied with patients who received PNB as compared to patients who received CB (p<0.002). Reduced venous engorgement with PNB presented a favorable intraoperative surgical site. Lower postoperative pain scores and reduced analgesic consumption in these patients also contributed to better surgeon satisfaction score in our study. Naja et al. [1] also observed similar findings when they compared PNB to CB.

In our study, the parents of three patients reported nausea, and two patients had vomiting in whom CB was given, while in those who received PNB, only two parents reported nausea in the postoperative period. No signs of hematoma or infection at the block site were observed. We did not encounter any major complications with both blocks. Wong et al. [3] had evaluated the implementation of enhanced recovery protocol in hypospadias repair surgeries and were successful in it. Postoperative pain is a major contributing factor in enhanced recovery after surgery; hence, the routine administration of PNB in patients undergoing hypospadias repair may allow early discharge from hospital.

The limitation of our study was that it was a prospective observational comparative study. Though the groups were equivalent, randomization and blinding were not done. We only observed immediate postoperative complications in both groups, but CB has been associated with the increased incidence of urethrocutaneous fistulae [4,9]. A prolonged follow-up of up to three months was required to evaluate the safety profile of both blocks. Recently, the ultrasound-guided technique of PNB has been described; our technique was nerve stimulation-guided PNB.

## Conclusions

We suggest that PNB can provide effective, longer-duration, and ideal postoperative analgesia for hypospadias surgery in children with fewer postoperative complications. However, further studies are required to evaluate the efficacy of PNB with USG and peripheral nerve stimulator, either individually or in combination.
